# Inferring Hypotheses on Functional Relationships of Genes: Analysis of the *Arabidopsis thaliana* Subtilase Gene Family

**DOI:** 10.1371/journal.pcbi.0010040

**Published:** 2005-09-23

**Authors:** Carsten Rautengarten, Dirk Steinhauser, Dirk Büssis, Annick Stintzi, Andreas Schaller, Joachim Kopka, Thomas Altmann

**Affiliations:** 1 Institut für Biochemie und Biologie, Genetik, Universität Potsdam, Golm, Germany; 2 Max-Planck-Institut für Molekulare Pflanzenphysiologie, Golm, Germany; 3 Institut für Physiologie und Biotechnologie der Pflanzen, Universität Hohenheim, Stuttgart, Germany; Ceres Inc., United States of America

## Abstract

The gene family of subtilisin-like serine proteases (subtilases) in *Arabidopsis thaliana* comprises 56 members, divided into six distinct subfamilies. Whereas the members of five subfamilies are similar to pyrolysins, two genes share stronger similarity to animal kexins. Mutant screens confirmed 144 T-DNA insertion lines with knockouts for 55 out of the 56 subtilases. Apart from SDD1, none of the confirmed homozygous mutants revealed any obvious visible phenotypic alteration during growth under standard conditions. Apart from this specific case, forward genetics gave us no hints about the function of the individual 54 non-characterized subtilase genes. Therefore, the main objective of our work was to overcome the shortcomings of the forward genetic approach and to infer alternative experimental approaches by using an integrative bioinformatics and biological approach. Computational analyses based on transcriptional co-expression and co-response pattern revealed at least two expression networks, suggesting that functional redundancy may exist among subtilases with limited similarity. Furthermore, two hubs were identified, which may be involved in signalling or may represent higher-order regulatory factors involved in responses to environmental cues. A particular enrichment of co-regulated genes with metabolic functions was observed for four subtilases possibly representing late responsive elements of environmental stress. The kexin homologs show stronger associations with genes of transcriptional regulation context. Based on the analyses presented here and in accordance with previously characterized subtilases, we propose three main functions of subtilases: involvement in (i) control of development, (ii) protein turnover, and (iii) action as downstream components of signalling cascades. Supplemental material is available in the Plant Subtilase Database (PSDB) (http://csbdb.mpimp-golm.mpg.de/psdb.html) , as well as from the CSB.DB (http://csbdb.mpimp-golm.mpg.de).

## Synopsis

The first complete plant genome sequence was available for *Arabidopsis thaliana,* a common weed. The number of genes in the *Arabidopsis* genome is estimated to be around 25,000. The functions of most of these gene are, however, still unknown. Many genes are grouped into gene families due to conserved sequences and predicted protein structures. In this article, the large subtilisin-like serine protease (subtilase) family of *Arabidopsis* is analysed. Although 56 subtilase genes have been identified in *Arabidopsis,* the function of only two subtilases is known. Analysis of mutants has revealed no further hints about the function of the other 54 subtilases. Here the authors present a novel approach to infer hypotheses about functions of the subtilase genes using computational analysis. Based on the analyses presented here and in accordance with previously characterized subtilases, they propose three main functions of subtilases: involvement in (i) control of development, (ii) protein degradation, and (iii) signalling. The results presented can be used to direct further analysis to elucidate functions of subtilases in plants.

##  Introduction

Subtilisin-like proteases (subtilases) are serine proteases with a catalytic triad of the three amino acids aspartate, histidine, and serine [1]. Eukaryotic subtilases belong to the S8 serine protease family (http://merops.sanger.ac.uk) and can be grouped into the pyrolysins and the kexins. Nine subtilases, the proprotein convertases, have been characterized in mammals. Of these, seven belong to the kexin subfamily, and two recently identified subtilases to the pyrolysin subfamily [2,3]. Kexin was identified as the first eukaryotic subtilase required in yeast for the processing of the precursors of α-mating factor and of killer toxin [4]. The seven mammalian kexin homologs are involved in the formation of peptide hormones, growth factors, neuropeptides, and receptor proteins from precursor polypeptides [2,3]. The two mammalian pyrolysins carry out specific cleavage and processing reactions on sterol regulatory elements, binding proteins, and pro-brain-derived neurotrophic factors, respectively [5,6]. The subtilase gene families in plants exceed in number that of mammalian subtilases by far [3]. They probably expanded to mediate a much wider range of processes. All hitherto identified plant subtilases have been grouped into the pyrolysin subfamily within the S8 serine protease family [7].

Despite the recent advances, our current understanding of subtilase functions in plants is still very limited. Currently, there is evidence for involvement of subtilases in both general protein turnover [8] and as highly specific regulation of plant development [9]. Few proteases have been purified from plant tissues and classified as subtilases based on their catalytic properties and primary structure [10–19]. Most of these enzymes are highly abundant and exhibit broad substrate specificity. Thus, a functional involvement in general protein turnover was forecasted for these abundant proteins [8,20,21]. The tomato subtilase P69 is one of several subtilases that are specifically induced following pathogen infection [22–24]. P69 processes a leucine-rich repeat cell wall protein in virus-infected tomato plants and thus is one of the very few plant subtilases for which an endogenous substrate has been identified [25]. The direct consequences of this processing event are still unknown. The P69 enzymes form a distinct subgroup among the 15 subtilases that have hitherto been cloned from tomato [26].

Forward genetics has identified subtilases as highly specific regulators of plant development. In the *Arabidopsis* SDD1 mutant (stomatal density and distribution 1), the pattern of stomata formation is disrupted, resulting in clustering of guard cells as well as in a dramatic increase of stomatal density [9]. The SDD1 gene is specifically expressed, and the protein is probably secreted into the apoplast [27]. Likewise, the gene disrupted in the ALE1 mutant (abnormal leaf shape 1) was cloned and found to encode a subtilase. ALE1 is required for cuticle formation and epidermal differentiation during embryo development in *Arabidopsis* [28]. The mutant phenotypes of SDD1 and ALE1 demonstrate that at least some subtilases carry out highly specific functions in plant development. Their modes of action in the regulation of the respective developmental processes are still unknown, but SDD1 and ALE1 may be required for the generation of peptide signals, which act non-cell autonomously to control plant development [27,28].

Despite the sequence homology–based prediction of 53 further *A. thaliana* subtilase (AtSBT) genes [29], there is still uncertainty about the functions of the majority of plant subtilases, including those of the model organism *Arabidopsis*. Here we describe results obtained with a complete set of gene knockout mutants and expression profile analysis. The main focus of our report is directed toward inference of hypotheses for functions of the so far uncharacterized *Arabidopsis* subtilase genes using computational analyses. We extended common classification of gene families by sequence similarity toward investigation into co-responding synchronous changes of transcript levels (co-response analyses). These generic data analysis procedures provided us with indications about the respective functional context of subtilases, which are presented here. These results demonstrate how the rapidly growing collections of gene expression profile data can be used to direct further experimental analyses to uncover gene functions.

## Results/Discussion

The goal of this work was to initiate the functional characterization of the *Arabidopsis* subtilase gene family members. A traditional entry point was based on pairwise or multiple sequence comparisons and alignments by various algorithms [30], which provides the means for functional prediction for genes or gene products by annotation transfer from homologous sequences [31,32]. We applied this approach to identify and classify *Arabidopsis* subtilase genes according to their sequence homology. However, initial attempts to transfer annotation did not provide us with strong clues to draw experimentally testable hypotheses due to the lack of characterized reference genes. Moreover, verified homozygous gene knockout lines revealed no obvious phenotypic alterations and, therefore, did not support basic functional assignment. Thus, gene expression co-response analysis was performed as an alternative procedure to generate hypotheses on functional contexts of *Arabidopsis* subtilases.

### The AtSBT Family Comprises 56 Genes

Our initial effort to identify subtilases was based on sequence comparisons with known and well-characterized *Arabidopsis* subtilase genes. Subtilases contain a catalytic triad (S8 domain) of the amino acid residues aspartate (Asp, D), histidine (His, H), and serine (Ser, S), as well as an asparagine (Asn, N), suggested to act as a substrate binding site. Sequence comparisons against AGI proteins (TAIR, [33]) were performed using the BLAST algorithm [34] with the S8 domain of the SDD1 amino acid sequence to identify homologous sequences. The identified sequences were evaluated for the presence of the conserved D-, H-, S-, and N-regions, and 56 subtilase encoding genes were detected (Table 1). Of the 56 genes, 55 encode proteins that contain all conserved motifs, while At5g45640 (AtSBT5.5) lacks the central Asp residue of the D-region. Hence, the subtilase family is among the largest protease gene families known in *Arabidopsis*. In addition to the previously identified 55 AtSBT genes [29], our analysis revealed another AtSBT containing the S8 domain, namely At4g20850 (AtSBT6.2).

**Table 1:**
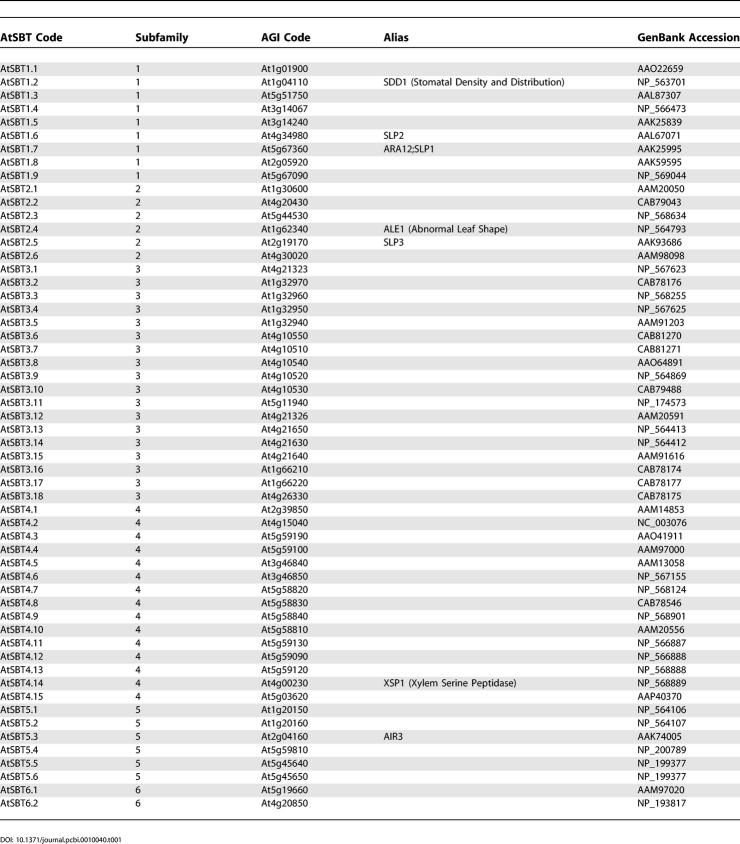
AtSBT Assignment and GenBank Accessions of the 56 Identified *Arabidopsis* Subtilase Genes

Beyond the sequence homology, predictions of the subcellular localization of a gene product provide additional indications for a possible functional involvement. Primary structure analysis using either TargetP [35] or PredoTar (http://genoplante-info.infobiogen.fr/predotar/predotar.html) indicated that most of the AtSBTs possess signal sequences for targeting to the secretory pathway. Six subtilases do not contain any known protein targeting motif. Three (one) family members are predicted to be targeted to mitochondria and one (three) to chloroplasts (Table 2). Experimental data for the subcellular localization of *Arabidopsis* subtilases are presently available only for SDD1 and for ARA12. In agreement with the predictions, they were both shown to be exported to the apoplast [19,27].

**Table 2:**
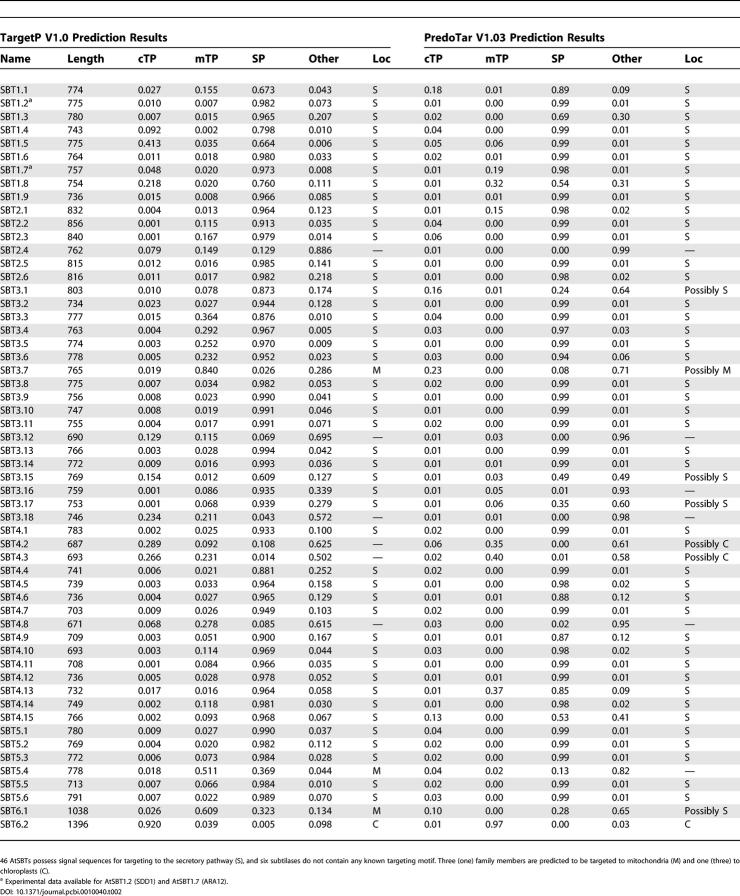
Targeting Prediction of the 56 *Arabidopsis* Subtilases using Either TargetP V1.0 or PredoTar V1.03

### The AtSBT Family Consists of Six Subfamilies

**Figure 1:**
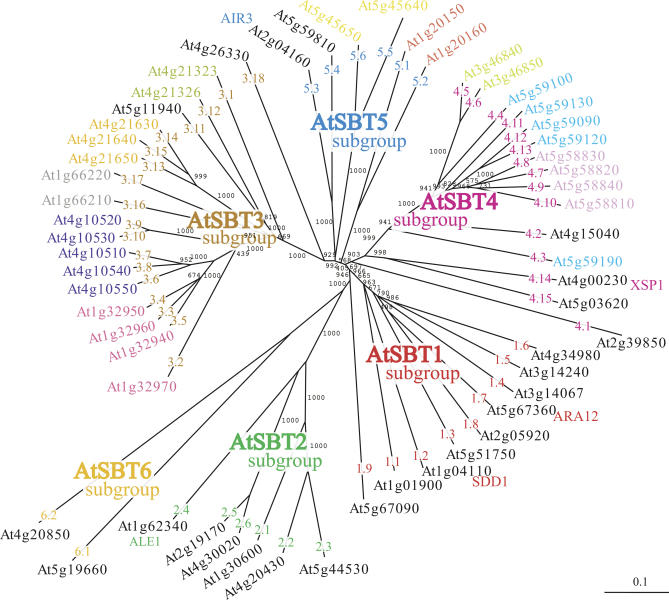
Bootstrapped Neighbour-Joining Tree Generated from an Alignment of the Predicted 56 AtSBT Full-Length Protein Sequences Groups of neighbouring genes (e.g., At3g46840 and At3g46850) are distinguished by specific colours.

Relationships of the 56 *Arabidopsis* subtilase sequences were investigated in order to identify pairs or groups of genes that could have overlapping or similar functions according to high similarities. We performed a multiple alignment with the deduced complete amino acid sequences. The obtained neighbour-joining tree, generated from this alignment, revealed six distinct subtilase subfamilies in *Arabidopsis* (Figure 1). The assignment of a gene to a specific subfamily was based primarily on the position within the phylogenetic tree, as defined by the homology between the deduced full-length amino acid sequences. When a gene could not be assigned to a particular clade with a significant bootstrap value, the assignment to a certain subfamily was made by ranking BLAST search results of queries for family members against the gene. Repeating the analysis by comparing only the conserved peptidase S8 domain, we could confirm the assignments for all *Arabidopsis* subtilase genes into the six subfamilies. The assignments were further supported by distance matrices obtained by pairwise global alignments of the nucleic acid (Table S1) and amino acid sequences (Table S2). The protease-associated (PA) domain is supposed to determine substrate specificities of subtilases or to form protein–protein interactions [36,37]. Most proteins of the subtilase family contain a sequence region of about 120 amino acids inserted into their catalytic domain. Therefore, to uncover similar substrate specificities within the *Arabidopsis* subtilase family, the PA domain was used for the assignment into subfamilies. Apart from AtSBT4.1, AtSBT 6.1, and AtSBT 6.2, all *Arabidopsis* subtilases contain an insertion consistent with a PA domain. Apart from members of the heterogeneous subfamily 5, all subtilases were again assigned to the same subfamilies as before with only minor changes (Figure S1).

The general consistency of phylogenetic trees derived from the full-length and the PA domain sequences suggests that the PA domain insertion was already present in ancestral subtilases. These results are consistent with those reported by Beers et al. [29], who defined three subgroups of *Arabidopsis* subtilases: S8–1, S8–2, and S8–3. The AtSBT1 and AtSBT2 subfamilies assembled through our analysis are identical to the S8–2 and S8–3 groups. The large heterogeneous S8–1 group, however, was subdivided further into AtSBT families 3, 4, and 5. The AtSBT6 subfamily includes only two members: i.e., AtSBT6.1, which had not been assigned to any group by Beers et al., and AtSBT6.2, which is a previously unrecognized *Arabidopsis* subtilase. Both genes are characterized by a stronger similarity to the mammalian kexins and pyrolysins than to plant subtilases, whereas all other *Arabidopsis* SBT subfamilies do not partition with any of the known human PCs (Figure S2). According to Killer toxin processing activity detected in plant extracts, the presence of plant kexin-like subtilases has been postulated [4,38]. In contrast to the mammalian kexin homologs required for the formation of functional proteins from precursor polypeptides [2,3], the identity of the plant kexin-like subtilases responsible for the observed processing reaction is unknown.

### Subtilase Families in Plants Exceed Complexity in Animals

Knowledge of phylogenetic relationships may help to unravel the basic functions of genes based on annotation transfer from orthologous sequences. BLAST searches using the peptidase S8 domain and several full-length amino acid sequences of *Arabidopsis* were performed via the NCBI *Oryza sativa* BLAST page [39] and revealed 34 non-redundant rice subtilase genes. A multiple sequence alignment of the 15 known tomato [26], the 34 identified rice, and the 56 *Arabidopsis* subtilases was performed to elucidate the phylogenetic relationships within the plant subtilase family. Within the obtained neighbour-joining tree, four major clusters of orthologous groups were identified that include all members of the AtSBT subfamilies 1–3 and 5, whereas AtSBT4 seems to be a subfamily specific for *A. thaliana*. The obtained neighbour-joining tree enabled us to identify putative orthologous pairs and groups of genes (Figure S3). However, the lack of functionally characterized orthologues in the subtilase family among the three plants species gave us no strong hints for functional annotation. Interestingly, all three plant species are characterized by significantly larger numbers of subtilases as present in animal organisms, e.g., human (nine), *Caenorhabditis* (four), or *Drosophila* (three), according to BLAST searches. The large number of *Arabidopsis* subtilase genes is the result of multiple duplication events (see below). Depending on the degree of functional diversification that occurred in the further evolution after the duplications, members of the gene family may have overlapping (“redundant”) or separate functions.

### Chromosomal Distribution and Gene Duplications of the AtSBTs

**Figure 2:**
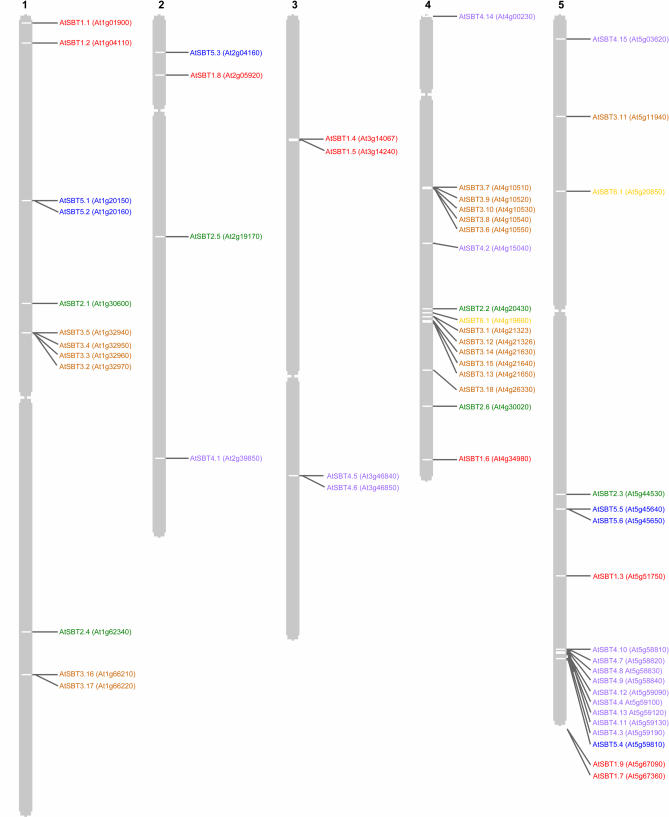
Physical Map of AGI at TAIR Indicating the Chromosomal Distribution of the AtSBT Family The AtSBT genes are localized throughout the *Arabidopsis* genome as single genes or in tandem repeats.

To unravel possible redundancy, we investigated the chromosomal locations of the AtSBTs and inferred the gene duplication events that probably caused this distribution. *Arabidopsis* subtilase genes are present on all five chromosomes (Figure 2). The genes occur isolated or in tandem repeats, indicating that segmental and tandem duplication events may have contributed to the evolution of the *Arabidopsis* subtilase gene family. In contrast to the observed average of 17% on genome scale [40], 54% of AtSBT genes, belonging only to the subfamilies 3, 4, and 5, occur in tandem duplications of two up to five genes. These arrangements suggest that local duplications events also contributed to the AtSBT family expansion. Furthermore, several highly similar sequences are found on different chromosomes. Similar situations indicative of a complex evolutionary history have been observed in other *Arabidopsis* gene families, too [41,42]. To determine whether the formation of the AtSBT gene family is in part the result of genome duplication events, we analysed the chromosomal distribution of the AtSBTs using the Genome and Redundancy Viewer (http://mips.gsf.de/proj/thal/db/gv/rv/rv_frame.html). Analyses of the chromosomal distribution revealed that at least 18 AtSBT genes are located in previously documented segmental duplicated regions within the *Arabidopsis* chromosomes.

Macro-scale duplication and rearrangement of chromosomes as well as micro-scale translocation and duplication are thought to be the major modes of plant genome evolution [43].

The results confirm local and segmental duplication events as the cause for expansion of the subtilase gene family in the course of the *Arabidopsis* genome evolution. As the two copies of a duplicated gene were initially identical and functionally redundant, the structure of the subtilase gene family poses the question: to what extent did the divergence of duplicated genes lead to the acquisition of novel and specific functions of subtilases in *Arabidopsis?*

### Mutant Identification and Evaluation

To elucidate the functions of all *Arabidopsis* subtilases, T-DNA insertion mutants have been collected and analyzed for morphological traits. A total of 179 obtained T-DNA insertion lines of 55 AtSBTs have been tested by PCR with gene-specific primers for the presence of the proposed insertion, which was confirmed in 144 lines. For 44 genes, more than one verified T-DNA line is available, and for 55 AtSBT genes homozygous T-DNA insertion lines have been isolated (Table S3). Aerial organs of all homozygous lines were visually and microscopically examined at several developmental stages. Except for AtSBT1.2 (sdd1), no visible phenotypic alterations linked to the insertion were detectable. These observations suggest that either most AtSBT genes mediate specific, conditional responses, or, alternatively, that a large degree of functional redundancy exists among or within subsets of the subtilase family. Indications for the latter possibility were obtained by sequence analyses that identified groups or pairs of closely related genes (see above). To test for potential homology-based functional redundancies, we created and confirmed double knockouts and knockout/RNAi lines (see PSDB). However, none of the obtained transgenic lines exhibited any morphological phenotypic alterations. While further in-depth analysis will be necessary, including monitoring of the responses to various environmental challenges and investigation of metabolic perturbations to complete the phenotypic characterization, these observations may indicate that (partial) functional redundancy may exist even among more family members showing higher sequence divergence. In order to obtain further indications as to which pairs or groups of genes may perform similar or overlapping functions despite low degrees of sequence similarity, and what their physiological roles may be, gene expression co-response analyses were performed.

### AtSBT Co-Expression and Co-Response Analyses

The increasing number of publicly available expression profiles analyzed in the frame of specific experiments enables scientists to use and to re-analyze the data for certain different questions. We investigated in such a cross-experimental approach by computational analysis of the co-expression and co-response behaviour of subtilases using 123 gene expression profiles publicly available from NASCArrays [44] and 192 profiles of the AtGenExpress developmental series [45]. The expression profile data were generated using the Ath1 gene chip technology platform (Affymetrix), which contains specific oligonucleotides for 52 of the 56 annotated AtSBT genes. We focused initially only on the AtSBT genes to compare the expression within the subtilase family. This analysis was (i) first, performed using the qualitative attributes “present,” “marginal,” and “absent” of the array technology platform, (ii) then extended to quantitative values of (relative) expression levels, and (iii) third, widened to include all other genes, allowing us to assign subtilases to defined functional classes based on their co-response behaviour with functional classified non-subtilase genes. The goal of our computational expression analysis was to infer experimentally testable hypotheses regarding the (i) functional interplay of AtSBTs and (ii) the functional contexts in which theses gene may be embedded.

### Ubiquitous and Conditional Expression of AtSBTs Revealed by Co-Expression Analysis

**Figure 3:**
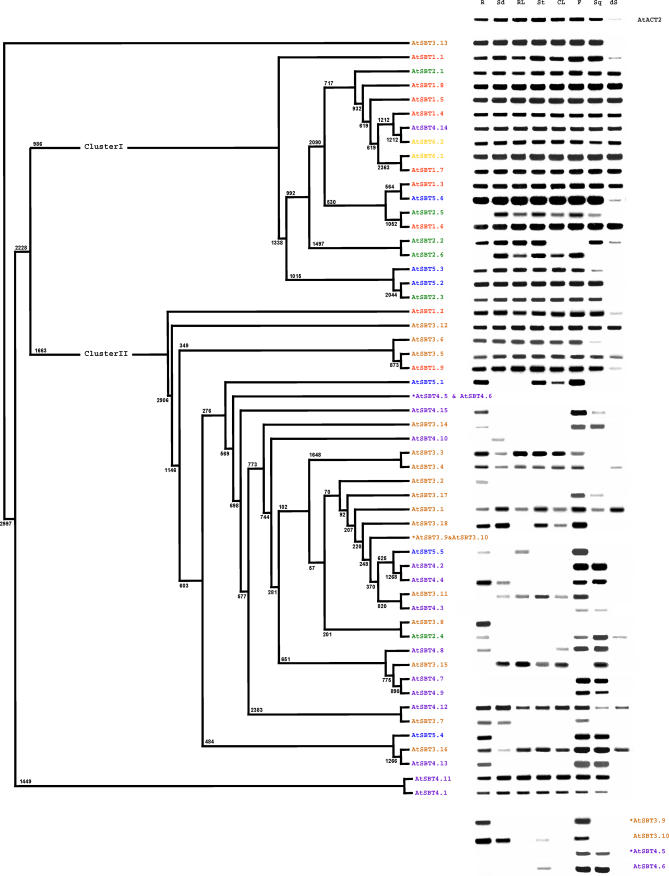
Bootstrapped Consensus Cluster Tree from Converted Detection Call Matrix of Affymetrix (Ath1) Microarray Experiments into Boolean Values (AtGenExpress Developmental Series) Cluster I covers ubiquitously expressed genes, whereas Cluster II mainly represents lowly or specifically expressed genes. These results were validated independently by semi-quantitative RT-PCR analysis (shown in the right panel). CL, cauline leaves; dS, dry seed; F, flower; R, root; RL, rosette leaves; Sd, seedling; Sq, siliques; St, inflorescence stem.

Our first computational investigation regarding the expression behaviour of subtilase genes focused on the grouping of AtSBTs according their coherent expression under identical experimental conditions. Coherent gene expression identifies either ubiquitous or conditional expression and allows first insight into a possible functional interplay of genes. To investigate the co-expression of AtSBTs, we converted the detection calls into qualitative Boolean values: (i) absent and marginal detection calls were set to null and (ii) present calls to one. Pairwise distances among all genes were computed using the S9 index via bootstrap analyses [46] with 999 numbers of bootstrapped Boolean matrices for each dataset (see Materials and Methods). The corresponding distance matrix was subjected to hierarchical cluster analysis (HCA) of the genes. Cluster trees drawn on the basis of bootstrap and non-bootstrap analyses revealed perfect agreement, i.e., the genes were assigned into the same cluster with same tree sorting. Validity and statistical significance of the clusters and the cluster tree structure were supported by bootstrap support values drawn on the basis of the resulting consensus cluster tree (see Materials and Methods). As a result of this analysis, we identified two most distantly related AtSBT gene clusters (Figure 3, Table 3, PSDB). Gene cluster I contained 18 (32%) of the 54 represented AtSBT genes and showed the following subfamily representation: AtSBT1: seven (78%), AtSBT2: five (83%), AtSBT3: zero (0%), AtSBT4: one (7%), AtSBT5: three (50%), AtSBT6: two (100%). In contrast, gene cluster II contained 35 (62%) AtSBT genes, which all belong to the subfamilies AtSBT1, AtSBT2, AtSBT3, AtSBT4, and AtSBT5. Whereas cluster I mainly represents ubiquitously expressed genes with some expressed at high levels, cluster II primarily contains genes with specific expression pattern and/or low expression levels. To confirm the obtained co-expression behaviour of AtSBTs and to test for biological relevance underlying the statistically significant clusters, we investigated their tissue-specific expression using semi-quantitative RT-PCR analyses. The obtained organ-specific expression patterns of the analyzed genes revealed ubiquitous expression for cluster I genes (Figure 3). The genes assigned to cluster II, on the other hand, exhibited expression primarily in one organ or in a subset of the analyzed organs. For some genes assigned to cluster II, namely AtSBT1.2, 1.9, 3.5, 3.6, and 3.12, we confirmed expression pattern for most of the analyzed organs. According to the results obtained by both analyses, we concluded that genes of cluster I are constitutively expressed, both in terms of organ specificity as well as according to various conditions. In contrast, genes of cluster II mainly show specific expression patterns (Figure 3).

**Table 3:**
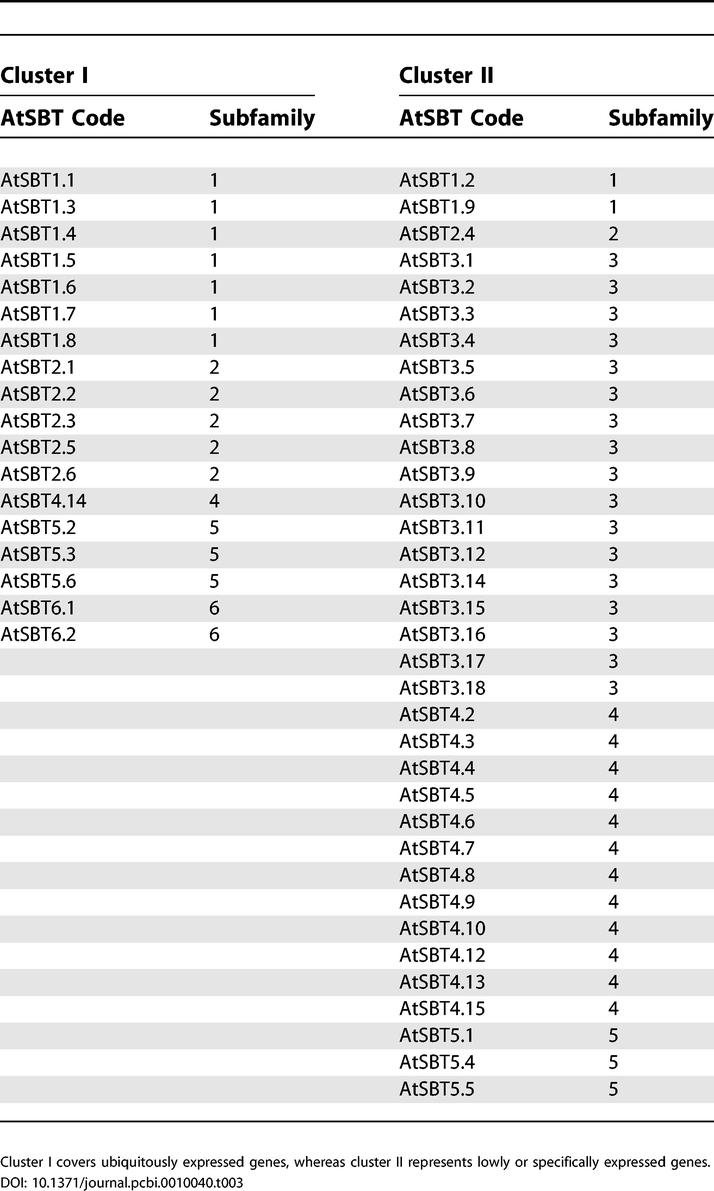
AtSBT Family Member Distribution within the Main Cluster I and II

### Transcriptional Interrelation among AtSBTs Revealed by Co-Response Analyses

While through the (qualitative) co-expression the global activity profiles of the AtSBT genes were revealed, the (quantitative) co-response analysis was performed to identify pairs or groups of AtSBT genes that show similar transcript changes among a multi-conditional set of expression. This analysis was carried out to test for overlapping and possible redundant functional interplays of AtSBTs regarding their expression behaviour. For our subsequent analyses, we implicitly make the assumption that common transcriptional control of genes is reflected in co-responding, simultaneous changes in transcript levels [47]. A necessary prerequisite for a co-response analysis is a considerable variation of gene expression levels across the datasets used. Furthermore, valid measures of expression, i.e., values above the detection limit, have to be available for the genes in question in most, ideally all, profiles. For the analysis, three multi-conditional gene expression data matrices (replicates) were assembled, each consisting of one of three replicates of approximately 50 expression profiles. The 50 selected datasets were composed approximately equally by a range of examined experimental condition (out of a total of 123 replicated experiments). These matrices were maximised for the diversity of the represented experimental conditions. Each of them covered series of valid gene expression values of approximately 10,000 genes, including 12 AtSBT genes, with valid measured transcript levels.

**Figure 4:**
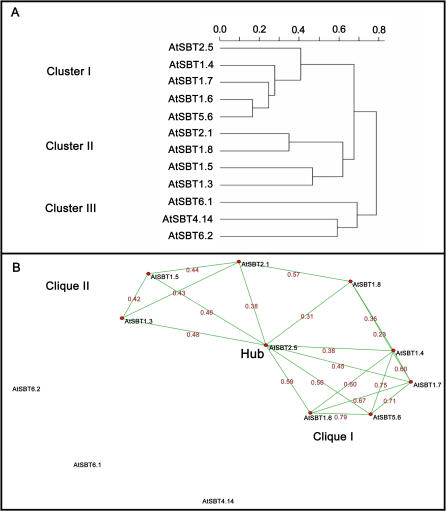
Transcript-Level Correlations of Ubiquitously Expressed Subtilase Genes in Multi-Conditional 22k Affymetrix Expression Profiles (A) shows a cluster tree of the of the transcript level correlations of ubiquitously expressed subtilase genes in multi-conditional 22k Affymetrix expression profiles. Correlations were determined by computing the Spearman's rank order correlation. (B) shows a network of all significantly positive associated AtSBTs according to the average Spearman correlation.

Our numerical approach to detect transcript co-responses is based on the non-parametric Spearman's rank order correlation (r_s_), which is a robust estimation of correlation. For bias estimation, as well as for a more exact approximation of the statistical probability, we performed iterative computation of r_s_ based on bootstrap analysis. A test of homogeneity was applied to compare the co-responses derived from the three data matrices and revealed no significant differences among the pairwise transcript co-responses. As the test of homogeneity can detect only large differences among pairwise transcript co-responses derived from different data matrices, we applied in addition the mantel test, performed as non-parametric Spearman correlation of matrices. The mantel test was used to estimate the association between the three independent data matrices describing the same set of entities. It tests whether the association between the matrices is stronger than a random association, i.e., whether similar transcript co-responses and similar biologically relevant information is coherent in the different matrices. This analysis showed highly significant correlations (*p* << 0.001) in the range of 0.87 ≤ r_s_ ≤ 0.90, with an average of 0.89 ± 0.02 among the matrices. Both statistical tests revealed that similar information about transcript co-responses could be deduced from the matrices. Therefore, the common transcript co-responses could be computed and used for HCA. Figure 4A shows a cluster tree drawn on the basis of the common co-responses for the three matrices. Spearman correlations (−1 < = r_s_ < = 1) were converted into distance measures (d) by the simple transformation d = 1−r_s_. Genes that showed opposing changes of transcript levels (with most negative co-responses) thus were displayed with largest distances, while the most highly correlated genes had smallest distances. The 12 represented AtSBT genes were grouped into three well-separated clusters: (i) with AtSBT2.5, AtSBT1.4, AtSBT1.7, AtSBT1.6, and AtSBT5.6, (ii) with AtSBT2.1, AtSBT1.8, AtSBT1.5, and AtSBT1.3, and (iii) with AtSBT6.1, AtSBT4.14, and AtSBT6.2. The joints were at relatively large heights and reflected that the corresponding changes in transcript levels were not identical but similar among pairs and groups of AtSBT genes. For further analysis, we visualized as a network significant, Bonferroni corrected [48] correlations among the AtSBT genes using the Pajek software [49] (Figure 4B). In conjunction with the cluster tree drawn on the basis of the common co-responses (Figure 4B), the obtained AtSBT network revealed two AtSBT cliques, where each gene member showed significant correlation to the other members. Clique I covered AtSBT1.4, AtSBT1.6, AtSBT1.7, AtSBT5.6, and AtSBT2.5, whereas the clique II included the genes AtSBT1.3, AtSBT1.5, AtSBT2.1, and AtSBT2.5. The subtilase AtSBT2.5 is shared between both cliques and represents a “hub” within the AtSBT network, which shows significant connections to all genes of the two main cliques and interconnects both AtSBT cliques (Figure 4). The average co-response of AtSBT2.5 to both cliques was 0.47 ± 0.08. Exclusion of AtSBT2.5 revealed an average co-response of 0.69 ± 0.08 within clique I and of 0.43 ± 0.01 within clique II. AtSBT1.8 is positively correlated with AtSBT2.5, but this gene shows less connectivity to the two cliques.

The statistical analyses revealed significant co-responses among AtSBT genes, but the causality of the interrelations remains to be shown. Non-parametric Kendall's tau (τ) correlation of *E. coli* operon genes controlled by common cis-elements revealed a co-response distribution over a broad range [47]. Considering the relationship of Spearman's r_s_ and Kendall's τ (r_s_~ 3/2 τ), the co-responses among AtSBT genes of clique I are in the upper range of these distributions. Therefore, a biological relevance of the observed co-response network can be assumed. In conjunction with the results of semi-quantitative RT-PCR and the co-expression analysis (see above and Figure 3), we conclude that the genes of clique I are ubiquitously but not constitutively expressed and that they respond to similar cues. The revealed associations and the central positions of AtSBT2.5 and AtSBT1.8 in the network suggest that both genes might be involved in the same functional context and may have (partially) overlapping roles. However, the similarities of the amino acid (32.9%) and the nucleic acid sequences (50.0%) of these two genes are not higher than their homologies to other AtSBT genes (average 34.7%/50.5%; see PSDB). In contrast, AtSBT2.5 is highly related to AtSBT2.6 (aa: 88.1%; nt: 83.7%), both are ubiquitously expressed, and have probably evolved from a sequential duplication. Redundancy of function that might be assumed according to the close evolutionary relationship was not supported, as a verified double homozygous T-DNA insertion line did not show any visible mutant phenotype despite their similar expression pattern (Figure 3). Similarly, AtSBT5.6 is highly related to AtSBT5.5 at the sequence level (aa: 62.3%; nt: 68.0%), but only AtSBT5.6 is a member of clique I. Sequence similarities between AtSBT5.6 and other members of clique I (average aa: 40.5%; average nt: 52.9%) are not notably higher than other AtSBT genes (average aa: 36.9%; average nt: 51.3%). In contrast, AtSBT1.4, AtSBT1.6, and AtSBT1.7 represent an example of evolutionary related genes with higher-than-average homology on the amino acid (46.8%–54.3%) and nucleic acid (57.7%–59.7%) level that are members of the clique I and show significant co-regulation. Nevertheless, AtSBTs with even higher sequence homology but lower co-response are present in subfamily 1. The co-response analysis of the AtSBT gene family thus revealed potential functional relationships, which in some cases clearly contradicted the predictions made on the basis of sequence analysis. In conclusion, we suggest that even minor differences in sequence similarity may confer functional divergence and that functional redundancy within the *Arabidopsis* subtilase family may be better revealed by transcriptional co-response analysis than by high sequence similarity. It is very likely that a few amino acid changes could alter the substrate specificity of a protease. A striking example of the consequences of a single amino acid change on the properties of an enzyme is provided by the stilbene synthases [50].

### Co-Response-Based Transcriptional Neighbourhood Search of AtSBTs

As a third step, we extended our co-response analyses to the characterization of the co-responses of AtSBT genes with all other genes represented in the underlying data matrices. This was performed to identify sets of co-regulated genes that are assigned to certain functional categories and may provide information on the functional context of individual or groups of AtSBT genes.

The degree of transcript co-responses may be influenced by the selection of the experiments used for generating the (multi-)conditional data matrices, and predictions based on nearest neighbours may be of equivocal nature. However, we assumed that the enrichment of transcriptionally correlated genes of a certain functional category should be a more robust marker of the functional context of a gene of interest. To obtain such indications for the AtSBT genes, we selected the top two percent of the strongest positive as well as negative correlated genes to each AtSBT gene. We computed the enrichment by adding up relative impacts (RIs) of the genes assigned to particular functional categories, where the gene-specific RI was defined as the reciprocal of the number of assignments of a gene to different categories. As reference, we calculated the enrichment as mentioned above over all genes represented in the underlying data matrices.

**Figure 5:**
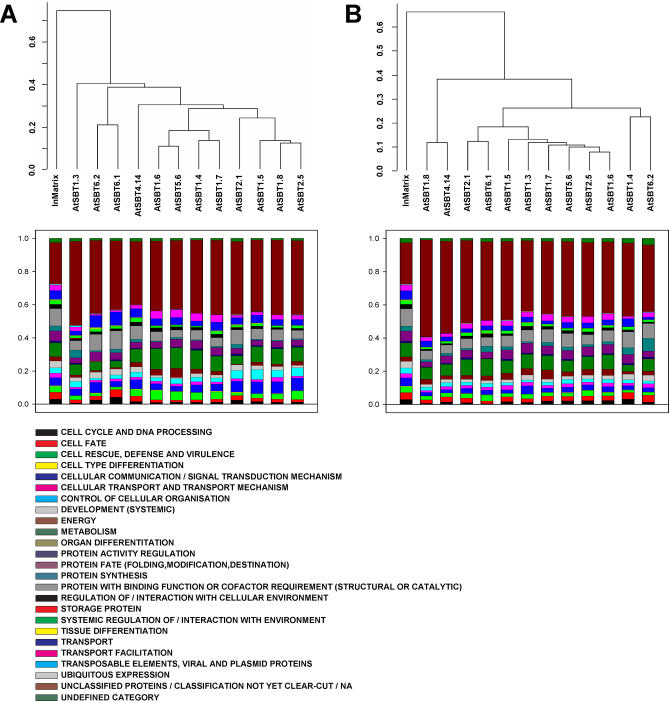
Result of the Co-Response-Based Transcriptional Neighbourhood Search for 12 Ubiquitously Expressed Subtilase Genes The best 2% of positively correlated (A) and of negatively correlated (B) genes were selected and used to determine functional category representations. The upper chart represents the cluster tree resulting from our HCA analysis based on conversion of the enrichment of genes of particular functional categories into the Euclidean distances. In the lower chart, vertically stacked bar plots illustrate the distribution of functional categories of correlated gene for each of the 12 AtSBTs. For comparison, the functional category distribution of the genes represented in the underlying data matrices is shown to the left of each display.

Applying the G-test of independence, which tests hypotheses about frequencies, for the positive best two percent correlated genes (Figure 5A) revealed that genes belonging to the category “unclassified” were significantly enriched (*p* << 0.001) for each of the 12 AtSBT genes, with an average of 1.78-fold. A significant (*p* < 0.05) enrichment of genes assigned to “metabolism” and “energy” was observed for AtSBT5.6. For AtSBT1.6, a member of the clique I (Figure 4), we detected a tendency (*p* < 0.1) of enrichment for “metabolism.” For AtSBT1.5, a member of the clique II (Figure 4), a significant enrichment for “control of cellular organisation” was observed. To categorise AtSBT genes according to their neighbourhood, we normalized each category-specific sum of RIs and expressed it as the fraction of the sum of all RIs over all categories. The co-responding matrix was subsequently used for hierarchical cluster analyses on the basis of the functional context in the neighbourhood by computing Euclidean distances. According to the obtained cluster tree for positive associated neighbourhood (Figure 5A), we suggested a similar functional context for AtSBT2.5, the major hub connecting cliques I and II (Figure 4B) and AtSBT1.8 that showed lower connectivity to the two cliques. Interestingly, analysis based on the two percent of strongly negative associated genes (Figure 5B) revealed different neighbourhoods for the two genes. According to these results, and consistent with the results of the co-expression (Figure 3) and co-response (Figure 4) analyses, we suggest that AtSBT2.5 and AtSBT1.8 have overlapping but not identical functions. The hub AtSBT2.5 and AtSBT1.8 are characterized by an enrichment of positively correlated genes assigned to “cellular communication/signal transduction mechanism” as well as “cellular organization,” which are ranked at positions 2 and 3.

Moreover, for the genes AtSBT1.4 and AtSBT1.7, as well as for AtSBT1.6 and AtSBT5.6, the members of clique I (Figure 4B), we observed early joining, according to the representation of functional classes by both the strongly positive and, with exception of AtSBT1.4, the strongly negative associated genes. According to a significant enrichment of genes assigned to the functional category “metabolism” (Figure 5A, PSDB), we suggest that these genes are embedded in the functional context of metabolism. These four genes were also correlated in expression with genes enriched for functions in “cell rescue, defence and virulence” and in “transport facilitation,” which are ranked at positions 3 and 4 (PSDB). The correlated behaviour and similar functional neighbourhoods of this set of AtSBTs hints to an involvement within the physiological context of pathogen response and/or general stress-related responses. The indications obtained for the functional contexts of these two sets of AtSBT genes leads us to suggest that AtSBT2.5 and AtSBT1.8 may be involved in sensing mechanisms, or might be early responsive factors. On the other hand, AtSBT1.4, AtSBT1.6, AtSBT1.7, and AtSBT5.6 may be related to more specific downstream processes. Such involvement in similar or identical processes may lead to similar transcriptional co-responses among genes as it was observed for the above-mentioned subtilases (Figure 4). Moreover, the HCA analyses of the neighbourhoods (Figure 5A) agreed with the tree and network drawn on the basis of the transcriptional interrelation among the ubiquitously expressed subtilases (Figure 4). The experimental verification of this hypothesis will be one of the goals of our continuing functional genomics project on the characterization of plant subtilases.

### The PSDB

Our research aims at the functional characterization of the subtilases in *Arabidopsis*. To achieve this goal, an international consortium of five European and US partners (The *Arabidopsis* Subtilase Consortium; http://csbdb.mpimp-golm.mpg.de/psdb.html) was established. The multiple levels of comprehensive data accumulated in this project by us and other groups within The *Arabidopsis* Subtilase Consortium need a specialized Web interface to store and distribute data related to plant subtilases. According to these needs, we established the PSDB, which is an associated database of the Comprehensive Systems-Biology Database (http://csbdb.mpimp-golm.mpg.de). PSDB contains confirmed results of replicated experiments related to plant *(Arabidopsis)* subtilase genes and allows open access to the science community. PSDB will be regularly updated with results of co-response analyses, performed on the increasing number of publicly available gene expression profiles. Furthermore, validated information of tissue-specific expression patterns of AtSBT genes, cellular localisation of encoded proteins, and phenotype information of the mutants and transgenic plants will be displayed and regularly updated. Further information and supplemental material will be available at PSDB (http://csbdb.mpimp-golm.mpg.de/psdb.html).

## Materials and Methods

### Sequence analysis.

Nucleic acid and amino acid sequences were retrieved by searching public databases with the BLAST algorithm [34] at TAIR (http://www.arabidopsis.org/), TIGR (http://www.tigr.org/), NCBI (http://www.ncbi.nlm.nih.gov), and MIPS (http://mips.gsf.de/). Subcellular localization was predicted using either TargetP V1.0 [35] (http://www.cbs.dtu.dk/services/TargetP/) or PredoTar V1.03 (http://genoplante-info.infobiogen.fr/predotar/predotar.html). The deduced amino acid sequences were aligned using the CLUSTALX program [51] with the default parameter settings and manually improved with respect to all known conserved subtilase motifs. The phylogenetic tree was obtained with the neighbour-joining method with bootstrap values generated from 1,000 bootstrap samples and visualized by using the TreeView application [52].

Only bootstrap values higher than 70% were considered to be significant [53]. Bootstrap values lower than 60% are not shown.

### Plant material and growth conditions.

Seeds of *A. thaliana* accessions Columbia-0 (Col-0), Wassilewskija, and the appropriate T-DNA mutant lines were surface-sterilized and germinated on half-concentrated Murashige and Skoog medium (M02 555 [pH 5.6], Duchefa, Haarlem, The Netherlands), supplemented with 1% sucrose, and solidified with 0.7% agar under a 16-h day (140 μmol·m−2·s−1, 22 °C)/8-h night (22 °C) regime. After 2 wk, plants were transferred to standard soil (Einheitserde GS90; Gebrueder Patzer, Sinntal-Jossa, Germany) and further grown in a growth chamber under a long-day light regime (16 h of fluorescent light [120 μmol·m−2·s−1] at 20 °C and 60% relative humidity/8 h of dark at 16 °C and 75% relative humidity).

### Mutant collection, confirmation, and phenotypic analysis.

T-DNA insertion mutants were retrieved from the SIGnAL [54], the GABI-Kat [55], the Genoplante FST/FLAGdb [56], the SAIL collection (Syngenta Biotechnology, Research Triangle Park, North Carolina, United States), and the University of Wisconsin Knockout facility. Genomic DNA was isolated using the DNeasy 96 Plant Kit (Qiagen, Hilden, Germany) and subsequently used for PCR analysis. The T-DNA insertion lines were screened for the appropriate insert using the required T-DNA and a gene-specific primer. Gene-specific flanking primers were used to confirm homozygosity. Primer sequences are available at PSDB. Homozygous insertion lines were evaluated for phenotypic alterations at the following developmental stages [57]: 1.03 for seedlings grown on synthetic media, 3.9 for rosette leaves, and 6.9 for inflorescence stem, cauline leaves, flower, and siliques. Plants were examined for leaf number, shape and size, epidermal constitution with respect to trichome and guard cell number and distribution, flowering time, and flower silique and seed morphology.

### Data source and pre-processing.

123 publicly available expression profiles from 22 experiments were obtained from NASCArrays (http://affymetrix.arabidopsis.info/, October 2003 [44]) and used for the generation of the data matrices nasc0271–0273. Additionally, 192 wildtype microarray profiles of 64 different tissues or developmental stages from the AtGenExpress developmental series generated at the Weigel laboratory have been used (http://www.arabidopsis.org/ [45]). The profiles were obtained through use of the Affymetrix Ath1 chip technology (Affymetrix, Santa Clara, California, United States), and results were scale normalized to TGT 100. The number of Present and Marginal calls (according to the MAS 5.0 algorithm) was calculated for each profile.

Transcript co-responses were retrieved from CSB.DB [58] for data matrix nasc0271. Co-responses for the additional matrices nasc0272 and nasc0273 were computed within this work (see below). In the majority of cases, two or three profiles per experiment with the highest numbers of Present and Marginal calls were selected for nasc0271. In analogy, nasc0272 and nasc0273 were generated from profiles per experiments ranked second and third according to the numbers of Present and Marginal calls. Thus, each of the data matrices comprised approximately 50 out of 123 profiles approximately equally representing the 22 underlying experiments, with approximately 10,000 out of more than 22,000 genes: nasc0271: 51 experiments with 9,694 genes, nasc0272: 51 experiments with 8,927 genes, and nasc0273: 49 experiments with 8,691 genes, each well measured in at least 85% of the underlying expression profiles. Transcript co-responses were computed on data matrices with log base 2 transformed and range-normalised transcript intensities for each gene.

### Co-expression analysis.

For co-expression analyses, the detection calls were converted into Boolean values. The profiles of the developmental series were separated into three data matrices according to the number of replicated experiments, whereas the NASC arrays are combined in one matrix. The numerical value null was assigned to absent and marginal calls, whereas present calls were set to one. Pairwise distances among entities, i.e., genes, of the Boolean matrix were computed using the S9 index via bootstrap analyses with 999 numbers of bootstrapped Boolean data matrices [46]. Each of the generated 999 pairwise distance matrices were subsequently used for HCA [59]. The computation was executed with the statistical software environment R2.1.0 [60]. HCA was performed as unweighted average linkage clustering algorithm. The resulting hierarchical cluster trees were converted into newick tree format with the function “hclust2phylog” of “ade4” package [60] implemented in the R software [61]. The resulting consensus tree was computed with the program “consensus” of the Phylogeny Inference Package [62] of all bootstrapped cluster trees, i.e., 2,997 trees represent the basis of the consensus tree of the developmental dataset.

### Semi-quantitative RT-PCR expression analysis.

Samples of the appropriate organs from *Arabidopsis* Col-0 plants were harvested at the stages used for mutant screening (see above). Total RNA was isolated with TRIzol reagent (Invitrogen, Carlsbad, California, United States) according to the manufacturer's protocol. 1 μg of total RNA was pre-treated with DNaseI (Ambion, Austin, Texas, United States) and reverse transcribed with SuperScriptII reverse trascriptase (Invitrogen) and d(T)_15_. The cDNA reaction was diluted 1:5 with water, and 5 μl of the diluted cDNA was used as template for PCR analysis applying Tit Taq PCR Enzyme System (BD Biosciences, Palo Alto, California, United States) according to the manufacturer's protocol. In general, due to the low abundance of subtilase transcripts, 40 cycles were performed with a PTC-200 thermal cycler (MJ Research, Waltham, Massachusetts, United States). Primer sequences and the size of cDNA and genomic amplicons are available via PSDB. AtACT2 was used as internal standard [63].

### Co-response analyses.

A general bivariate normality cannot be assumed for each gene pair, respectively analysed with the Cramer-test [64]; transcript co-response analyses were performed by computation of non-parametric Spearman's rank order correlation (r_s_) [65]. Co-response analysis among AtSBT genes were calculated by non-parametric bootstrap analyses with 2,000 numbers of bootstrap samples [66]. Mantel test and test on homogeneity [65] were used to compare and compute the common correlations among different co-response matrices. Testing homogeneity was performed using Microsoft Excel. The mantel test, computed as non-parametric Spearman correlation of (dis-)similarity matrices, was executed in R1.8.1. [61]. Common Spearman correlations and joint probabilities among the matrices were calculated as recommended [65]. In order to generate normalised distance matrices, correlations were converted into distance ranges [65]. Negative Spearman correlations were assigned to be most distant and converted into the largest distances: distance = 1 – r_s_. Normalization of the obtained distance matrix was done by dividing distances with the obtained maximum. HCA was performed as mentioned above. Visualization of significant associations was done with the software Pajek [49]. The multiple comparison performed required the adjustment of α to accept significant associations, which was done by application of the Bonferroni correction α′ = α/k. The corrected α′ for 12 comparisons was 0.00416.

### Transcriptional neighbourhood search.

The assignment of gene products to functional categories was retrieved from MAtDB (December 2003 [67]). The functional categorization is tree-like, and each category is further divided into subcategories. We used only the highest branch for each category, which included 99 categories, 29 assigned with a category name. Categories without category name were merged into the “undefined” category, and the categories 40, 43, 45, and 47 were merged into the class “localization.” Genes without assignment or with unclear classification were treated as “unclassified.” Genes assigned to more than seven categories, which represents 5% of the whole annotation, were also treated as “unclassified.” The RI of a gene with multiple assignments (n_assign_) onto each category was defined as ri = 1/n_assign_.

The transcriptional neighbourhood search was performed as follows: For each AtSBT gene, the best 2% of positively and negatively correlated genes to each represented AtSBT gene were extracted and grouped according to their assigned functional category. For calculation of the enrichment of functional categories, the sum of all RIs for each category was computed. As reference against which the enrichment/de-enrichment was determined, the sum of all RIs for each category over all represented genes was used. Significance of the observed category enrichments for each of the AtSBT was calculated for each functional category by G-test of independence [64].

## Supporting Information

Figure S1 Bootstrapped Neighbour-Joining Tree Generated from an Alignment of the Predicted PA Domain using Clustal X 1.81 The tree was displayed by TreeView and edited manually. Notice that out of 56 AtSBTs, three (6.1, 6.2, and 4.1) do not contain a PA domain.
(159 KB PDF)

Figure S2 Bootstrapped Neighbour-Joining Tree with 1,000 Bootstrap Replicates Generated from an Alignment of the Full-Length Protein Sequences of the AtSBT1, 2, and 6 Subfamily Members, Yeast Kex2p, and the Human Prohormone Convertases (PCs, Furin, SK1) using Clustal X 1.81 The tree was displayed by TreeView and edited manually.
(83 KB PDF)

Figure S3 Phylogenetic Tree of Plant *Arabidopsis,* Tomato, and Rice Subtilisin-Like Serine Proteases The Neighbour-Joining tree was generated from an alignment of the 56 AtSBT, 14 tomato (blue shaded), and 34 identified rice (black font) full-length protein sequences. Branch lengths are proportional to number of amino acid substitutions. Four major clusters of orthologous groups (MCOGs) were identified that included all members of the *Arabidopsis* subfamilies 1, 2, 3, and 5. The *Arabidopsis* subfamily 4 seems to be specific for this plant species, whereas with the exception of TMP, all analyzed tomato subtilases belong to the MCOG2.
(576 KB PDF)

Table S1 Distance Matrix Obtained by a Pairwise Global Alignment of the 56 AtSBT Nucleic Acid Sequences (46 KB XLS)

Table S2 Distance Matrix Obtained by a Pairwise Global Alignment of the 56 AtSBT Full-Length Amino Acid Sequences (46 KB XLS)

Table S3 Table of the T-DNA Insertion Mutants Collected and Tested by PCR with Gene-Specific Primers for the Presence of the Proposed Insertion and Analyzed for Morphological Traits (11 KB PDF)

## Abbreviations

AtSBT: *Arabidopsis thaliana* subtilase
HCA: hierarchical cluster analysis
PA: protease-associated
PSDB: Plant Subtilase Database
RI: relative impact
