# Supplementary material for: Inferring Hypotheses on Functional Relationships of Genes: Analysis of the Arabidopsis thaliana Subtilase Gene Family
Source: PLoS Comput Biol. 2005 Sep 23;1(4):e40. doi: 10.1371/journal.pcbi.0010040 (PMC1236819; doi:10.1371/journal.pcbi.0010040)
Supplement: Supplementary file 1 [file pcbi.0010040.sg001.pdf]

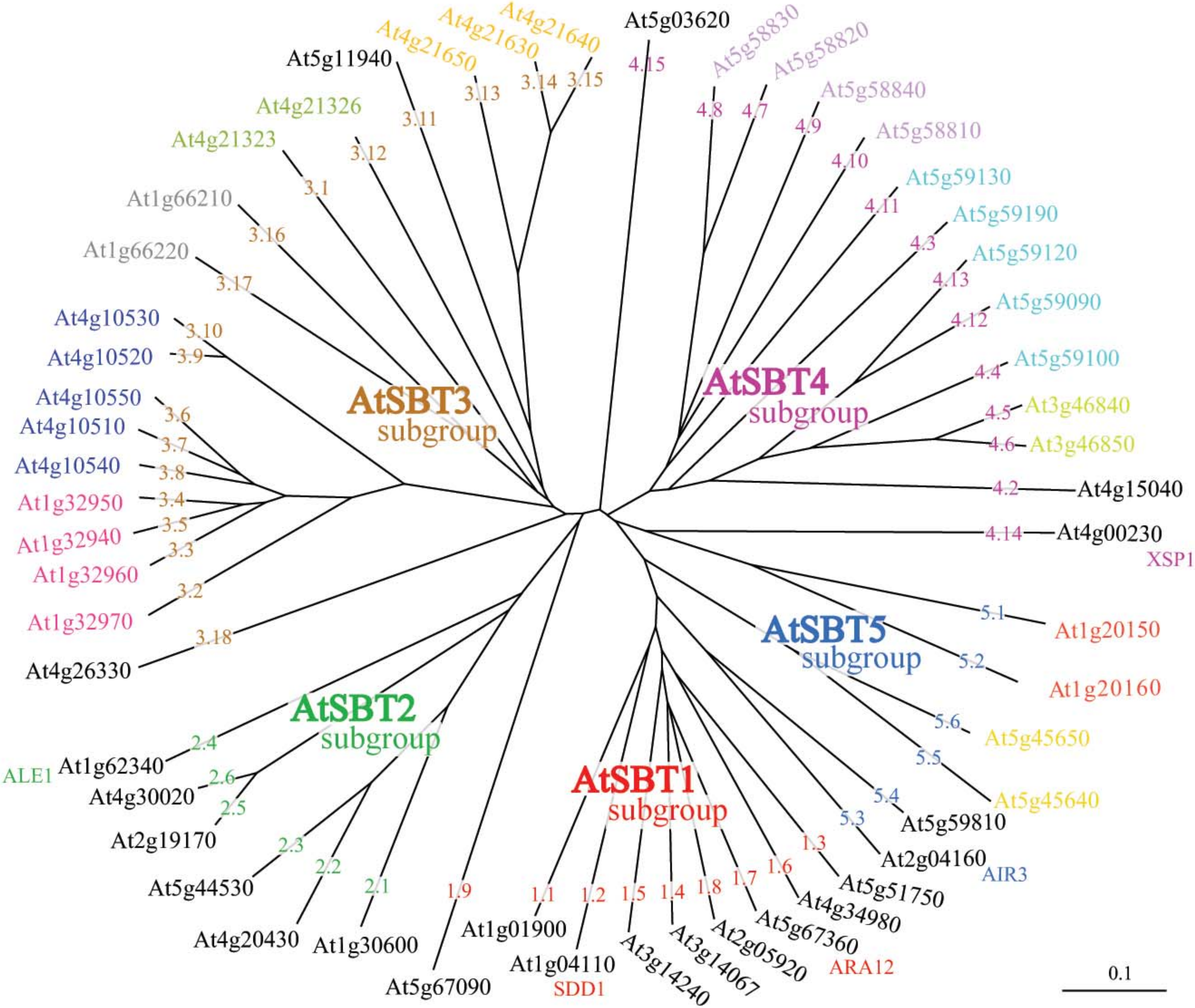

AtSBT1 subgroup
AtSBT2 subgroup
AtSBT3 subgroup
AtSBT4 subgroup
AtSBT5 subgroup
At5g03620
4.15
At5g11940
3.11
At4g21650
3.13
At4g21630
At4g21640
3.14
3.15
At4g21326
At4g21323
3.12
3.1
At1g66210
3.16
At1g66220
3.17
At4g10530
3.10
At4g10520
3.9
At4g10550
3.6
At4g10510
3.7
At4g10540
3.8
At1g32950
3.4
At1g32940
3.5
At1g32960
3.3
At1g32970
3.2
At4g26330
3.18
At5g58830
4.8
At5g58820
4.7
At5g58840
4.9
At5g58810
4.10
At5g59130
4.11
At5g59190
4.3
At5g59120
4.13
At5g59090
4.12
At5g59100
4.4
At3g46840
4.5
At3g46850
4.6
At4g15040
4.2
At4g00230
4.14
XSP1
At1g20150
5.1
At1g20160
5.2
At5g45650
5.6
At5g45640
5.5
At5g59810
5.4
At2g04160
5.3
AIR3
ALE1
At1g62340
2.4
At4g30020
2.6
At2g19170
2.5
At5g44530
2.3
At4g20430
2.2
At1g30600
2.1
At5g67090
1.9
At1g01900
1.1
At1g04110
1.2
SDD1
At3g14240
1.5
At3g14067
1.4
At2g05920
1.8
At5g67360
1.7
ARA12
At4g34980
1.6
At5g51750
1.3
0.1
