# Supplementary material for: Inferring Hypotheses on Functional Relationships of Genes: Analysis of the Arabidopsis thaliana Subtilase Gene Family
Source: PLoS Comput Biol. 2005 Sep 23;1(4):e40. doi: 10.1371/journal.pcbi.0010040 (PMC1236819; doi:10.1371/journal.pcbi.0010040)
Supplement: Supplementary file 2 [file pcbi.0010040.sg002.pdf]

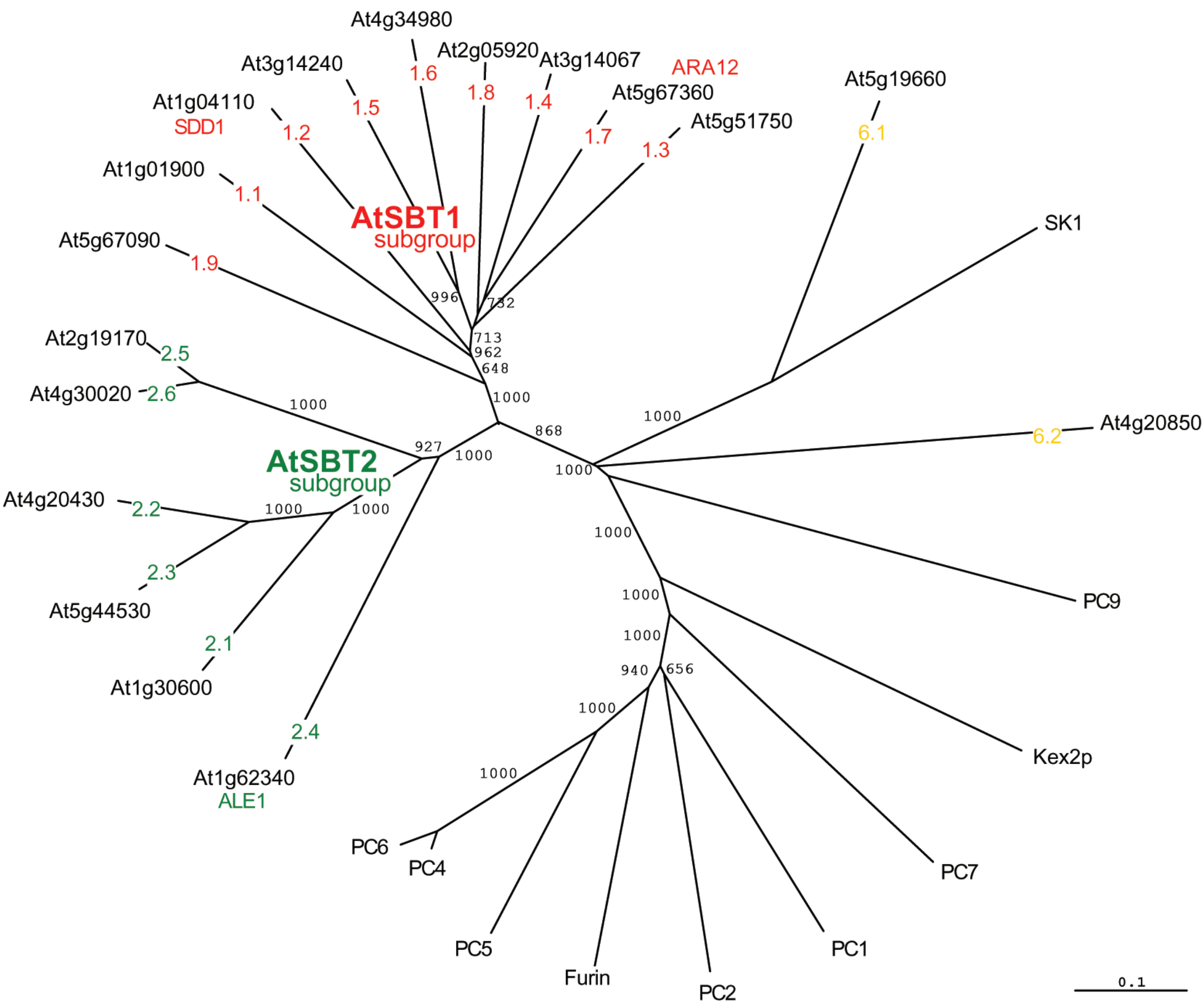
At4g34980
1.6
At3g14240
1.5
At2g05920
1.8
At3g14067
1.4
ARA12
At5g67360
1.7
At5g51750
1.3
At1g04110
SDD1
1.2
At1g01900
1.1
AtSBT1
subgroup
At5g67090
1.9
996
732
713
962
648
1000
At2g19170
2.5
At4g30020
2.6
1000
927
AtSBT2
subgroup
1000
868
1000
At4g20430
2.2
1000
1000
2.3
At5g44530
2.1
At1g30600
2.4
At1g62340
ALE1
At5g19660
6.1
SK1
1000
6.2
At4g20850
1000
1000
PC9
1000
1000
940
656
Kex2p
1000
1000
PC6
PC4
PC5
Furin
PC2
PC1
PC7
0.1
