# Supplementary material for: Inferring Hypotheses on Functional Relationships of Genes: Analysis of the Arabidopsis thaliana Subtilase Gene Family
Source: PLoS Comput Biol. 2005 Sep 23;1(4):e40. doi: 10.1371/journal.pcbi.0010040 (PMC1236819; doi:10.1371/journal.pcbi.0010040)
Supplement: Supplementary file 3 [file pcbi.0010040.sg003.pdf]

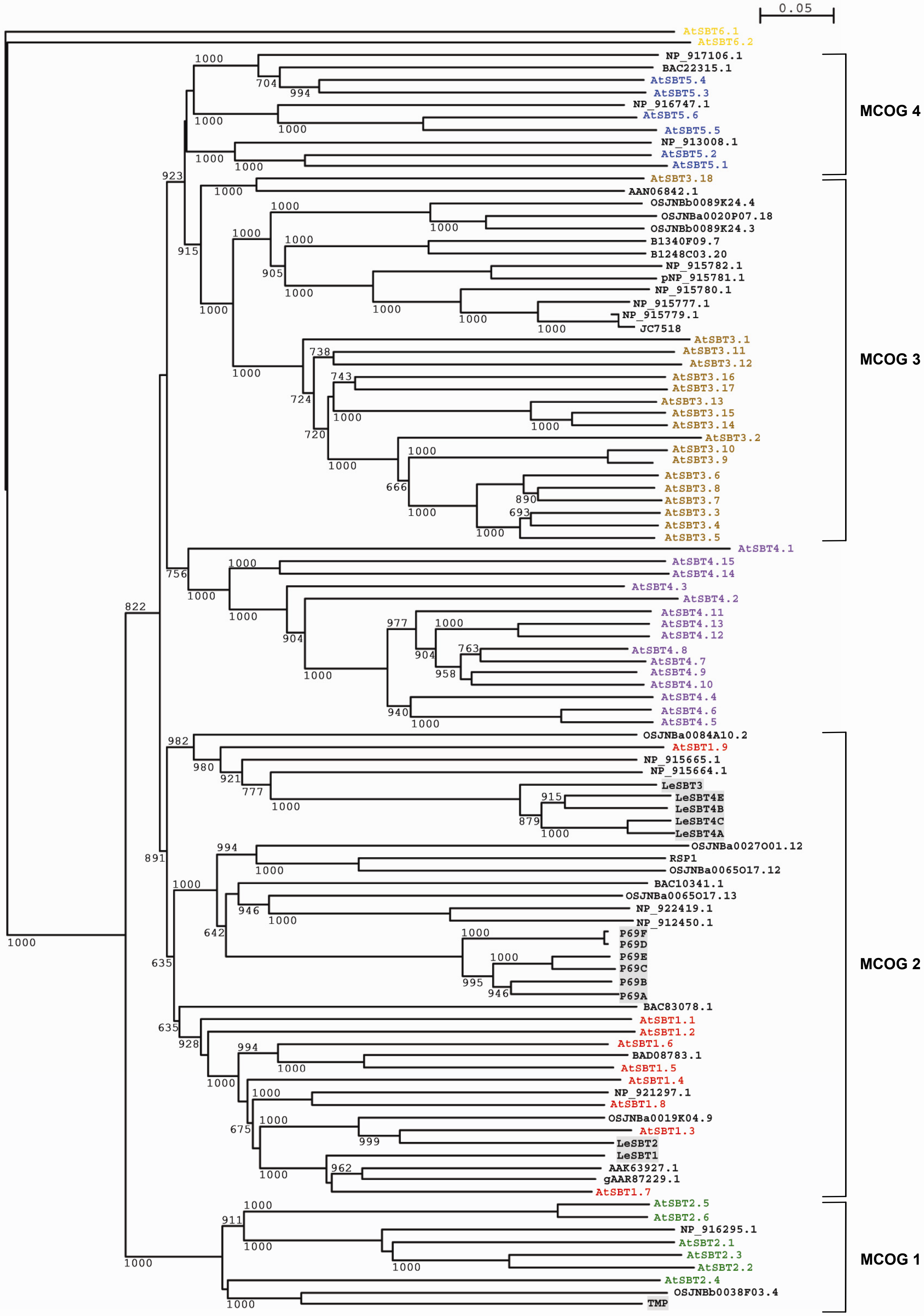
0.05
AtSBT6.1
AtSBT6.2
NP_917106.1
BAC22315.1
AtSBT5.4
AtSBT5.3
NP_916747.1
AtSBT5.6
AtSBT5.5
NP_913008.1
AtSBT5.2
AtSBT5.1
AtSBT3.18
AAN06842.1
OSJNBb0089K24.4
OSJNBa0020P07.18
OSJNBb0089K24.3
B1340F09.7
B1248C03.20
NP_915782.1
pNP_915781.1
NP_915780.1
NP_915777.1
NP_915779.1
JC7518
AtSBT3.1
AtSBT3.11
AtSBT3.12
AtSBT3.16
AtSBT3.17
AtSBT3.13
AtSBT3.15
AtSBT3.14
AtSBT3.2
AtSBT3.10
AtSBT3.9
AtSBT3.6
AtSBT3.8
AtSBT3.7
AtSBT3.3
AtSBT3.4
AtSBT3.5
AtSBT4.1
AtSBT4.15
AtSBT4.14
AtSBT4.3
AtSBT4.2
AtSBT4.11
AtSBT4.13
AtSBT4.12
AtSBT4.8
AtSBT4.7
AtSBT4.9
AtSBT4.10
AtSBT4.4
AtSBT4.6
AtSBT4.5
OSJNBa0084A10.2
AtSBT1.9
NP_915665.1
NP_915664.1
LeSBT3
LeSBT4E
LeSBT4B
LeSBT4C
LeSBT4A
OSJNBa0027O01.12
RSP1
OSJNBa0065O17.12
BAC10341.1
OSJNBa0065O17.13
NP_922419.1
NP_912450.1
P69F
P69D
P69E
P69C
P69B
P69A
BAC83078.1
AtSBT1.1
AtSBT1.2
AtSBT1.6
BAD08783.1
AtSBT1.5
AtSBT1.4
NP_921297.1
AtSBT1.8
OSJNBa0019K04.9
AtSBT1.3
LeSBT2
LeSBT1
AAK63927.1
gAAR87229.1
AtSBT1.7
AtSBT2.5
AtSBT2.6
NP_916295.1
AtSBT2.1
AtSBT2.3
AtSBT2.2
AtSBT2.4
OSJNBb0038F03.4
TMP
MCOG 4
MCOG 3
MCOG 2
MCOG 1
