# Supplementary material for: Inferring Hypotheses on Functional Relationships of Genes: Analysis of the Arabidopsis thaliana Subtilase Gene Family
Source: PLoS Comput Biol. 2005 Sep 23;1(4):e40. doi: 10.1371/journal.pcbi.0010040 (PMC1236819; doi:10.1371/journal.pcbi.0010040)
Supplement: Supplementary file 4 [file pcbi.0010040.st003.pdf]

AGI Code	AtSBT code	Mutants	FST position	Homozygous	Heterozygous	negative
At1g01900	AtSBT1.1	SALK_033704	exon	+		
		SALK_017912	exon	+		
		SALK_016856	exon			+
		Garlic_587_G07	exon	+		
		SALK_111561	exon	+		
At1g04110	AtSBT1.2	SALK_035559	exon	+		
		SALK_035560	5´			+
		Garlic_698_A07	5´	+		
At1g20150	AtSBT5.1	SALK_121716	5´	+		
		Garlic_06_E03	intron	+		
		SALK_017993	exon	+		
		SALK_121714	5'	+		
At1g20160	AtSBT5.2	SALK_012112	exon	+		
		SALK_012113	exon	+		
At1g30600	AtSBT2.1	GABI_202H08	exon	+		
		SALK_091134	exon	+		
		Garlic_638_A10	intron	+		
At1g32940	AtSBT3.5	SALK_106869	1000 p	+		
		Garlic_400_F09	exon	+		
At1g32950	AtSBT3.4	SALK_040245	exon	+		
		SALK_058032	intron	+		
		Garlic_888_D05	intron	+		
		SALK_013864	5'	+		
At1g32960	AtSBT3.3	SALK_086092	exon	+		
		SALK_107460	exon	+		
		Garlic_904_H05	exon	+		
At1g32970	AtSBT3.2	SALK_001743	1000 p	+		
		SALK_118300	3'			+
At1g62340	AtSBT2.4	Garlic_736_D09	intron	+		
At1g66210	AtSBT3.16	SALK_009433	exon	+		
		SALK_009498	exon			+
		SALK_004740	exon			+
		SALK_004741	exon	+		
		SALK_004739	exon	+		
		SALK_091136	exon	+		
At1g66220	AtSBT3.17	SALK_040473	exon	+		
		SALK_070765	exon	+		
At2g04160	AtSBT5.3	SALK_051293	intron	+		
		SALK_051199	intron			+
At2g05920	AtSBT1.8	GABI_168E04	exon	+		
		SALK_020799	exon	+		
At2g19170	AtSBT2.5	FLAG_181B06	exon	+		
		GABI_243B02	exon	+		
At2g39850	AtSBT4.1	SALK_016756	intron	+		
		SALK_038521	exon	+		
		FLAG_152A06	exon	+		
		SALK_053940	exon		+	
		SALK_138776	exon	+		
At3g14067	AtSBT1.4	SALK_054778	exon	+		
		SALK_063823	exon	+		
		SALK_004143	3´			+
		SALK_022941	exon			+
		SALK_118286	exon			+
At3g14240	AtSBT1.5	SALK_032651	exon	+		
		SALK_032650	exon			+
At3g46840	AtSBT4.5	GABI_251C03	exon	+		
		SALK_078286	intron	+		
		Garlic_87_F5	exon	+		
At3g46850	AtSBT4.6	SALK_091683	1000 p	+		
		WiscDsLox 304G08	exon	+		
At4g00230	AtSBT4.14	SALK_019254	exon	+		
		SALK_004888	3´	+		
		SALK_004983	3´		+	
		SALK_004887	3´			+
		SALK_069370	intron	+		
At4g10510	AtSBT3.7	SALK_081645	exon	+		
		SALK_099407	exon	+		
At4g10520	AtSBT3.9	SALK_048279	intron	+		
		SALK_074265	intron	+		
		SALK_048271	intron			+
		SALK_048262	intron			+
At4g10530	AtSBT3.10	SALK_014429	intron	+		
		SALK_035577	intron			+
At4g10540	AtSBT3.8	GABI_226F04	exon	+		
		SALK_052039	exon	+		
		SALK_142870	exon	+		
At4g10550	AtSBT3.6	SALK_104806	intron	+		
At4g15040	AtSBT4.2	SALK_024853	exon	+		
		WiscDsLox 393_396B20	intron	+		
At4g20430	AtSBT2.2	SALK_006217	3´	+		
		SALK_150020	exon		+	
		SALK_013152	exon		+	
		SALK_075830	exon			+
		SALK_075832	exon	+		
		Garlic_189_A11	exon	+		
At4g20850	AtSBT6.2	SALK_085776	exon	+		
At4g21323	AtSBT3.1	GABI_069E04	exon	+		
		Garlic_863_D02	exon			+
		SALK_062717	exon	+		
At4g21326	AtSBT3.12	SALK_037231	exon	+		
		SALK_130349	exon	+		
		SALK_130445	exon			+
At4g21630	AtSBT3.14	SALK_127987	exon	+		
		Garlic_410_G07	exon	+		
		SALK_036863	exon			+
		SALK_087162	exon			+
At4g21640	AtSBT3.15	SALK_012574	5´	+		
		SALK_040772	intron	+		
		SALK_064593	exon	+		
		SALK_137482	intron			+
		SALK_147913	intron			+
		SALK_063268	exon	+		
At4g21650	AtSBT3.13	SALK_082160	exon	+		
		SALK_020994	exon			+
At4g26330	AtSBT3.18	SALK_037331	3´	+		
		GABI_360C11	exon	+		
		SALK_110146	exon	+		
		SALK_119062	exon		+	
At4g30020	AtSBT2.6	GABI_125A08	exon	+		
		SALK_068944	exon	+		
At4g34980	AtSBT1.6	SALK_010591	3´	+		
		SALK_010879	3´			+
		GABI_270F06	exon		+	
		SALK_054228	exon	+		
		Garlic_435_G6	exon	+		
		SALK_090769	5'	+		
		SALK_080446	exon			+
At5g03620	AtSBT4.15	SALK_063258	exon	+		
		SALK_046549	intron			+
		SALK_097473	intron			+
		SALK_075714	exon			+
At5g11940	AtSBT3.11	SALK_035474	5´	+		
		SALK_067858	exon	+		
		SALK_007362	exon			+
At5g19660	AtSBT6.1	SALK_111474	exon	+		
		SALK_020530	exon	+		
		WiscDsLox 390C11	exon	+		
At5g44530	AtSBT2.3	GABI_081A06	intron	+		
		SALK_022324	exon	+		
At5g45640	AtSBT5.5	FLAG_136B03	intron	+		
		SALK_044318	intron		+	
		SALK_107233	exon	+		
		Garlic_209_A09	intron	+		
		SALK_049994	exon	+		
		WiscDsLox 468A6	exon			+
At5g45650	AtSBT5.6	GABI_074B04	intron	+		
		SALK_136178	exon		+	
		SALK_036514	intron			+
		SALK_017244	intron	+		
At5g51750	AtSBT1.3	SALK_011867	exon		+	
		SALK_123302	exon	+		
		SALK_141425	5'	+		
At5g58810	AtSBT4.10	SALK_119237	exon	+		
		GABI_389A08	exon	+		
		SALK_147509	exon	+		
At5g58820	AtSBT4.7	SALK_013603	exon	+		
		Garlic_26_G02	exon	+		
At5g58830	AtSBT4.8					
At5g58840	AtSBT4.9	SALK_060155	exon	+		
		Garlic_216_B07	exon	+		
		SALK_052517	exon			+
At5g59090	AtSBT4.12	GABI_239B04	exon	+		
		SALK_045125	exon		+	
		SALK_056823	exon	+		
		WiscDsLox 392E07	exon	+		
At5g59100	AtSBT4.4	SALK_016547	1000 p	+		
		SALK_018862				+
At5g59120	AtSBT4.13	SALK_009191	intron	+		
		GABI_230A02	exon	+		
		Garlic_228_A04	exon	+		
		SALK_103988	exon		+	
At5g59130	AtSBT4.11	GABI_132H10	exon		+	
		SALK_079334				+
		SALK_089292	exon	+		
		SALK_145867	exon	+		
At5g59190	AtSBT4.3	SALK_149055	exon	+		
		SALK_075909	exon	+		
		SALK_029933	3´		+	
		SALK_041189	intron		+	
At5g59810	AtSBT5.4	GABI_099F02	exon	+		
		FLAG_055C04	intron	+		
		SALK_072588	intron	+		
		SALK_150825	intron	+		
		SALK_025087	exon			+
At5g67090	AtSBT1.9	SALK_009925	exon		+	
		SALK_009917	exon	+		
		GABI_100G11	3´	+		
		SALK_009928				+
		Garlic_513_E02	exon	+		
At5g67360	AtSBT1.7	GABI_140B02	exon	+		
